# Heterogeneity of Clinical Presentations and Paraclinical Explorations to Diagnose Disseminated Histoplasmosis in Patients with Advanced HIV: 34 Years of Experience in French Guiana

**DOI:** 10.3390/jof6030165

**Published:** 2020-09-08

**Authors:** Mathieu Nacher, Audrey Valdes, Antoine Adenis, Romain Blaizot, Philippe Abboud, Magalie Demar, Félix Djossou, Loïc Epelboin, Kinan Drak Alsibai, Caroline Misslin, Balthazar Ntab, Pierre Couppié

**Affiliations:** 1 CIC INSERM 1424, Centre Hospitalier Andree Rosemon Cayenne, 97300 Cayenne, French Guiana; antoine.adenis@ch-cayenne.fr; 2 Département Formation Recherche Santé, Université de Guyane, 97300 Cayenne, French Guiana; romain.blaizot@ch-cayenne.fr (R.B.); pierre.couppie@ch-cayenne.fr (P.C.); 3 Equipe Opérationnelle d’Hygiène Hospitalière, Centre Hospitalier Andree Rosemon Cayenne, 97300 Cayenne, French Guiana; audrey.valdes@ch-cayenne.fr; 4 Department of Dermatology, Centre Hospitalier Andree Rosemon Cayenne, 97300 Cayenne, French Guiana; 5 Service des Maladies Infectieuses et Tropicales, Centre Hospitalier Andree Rosemon Cayenne, 97300 Cayenne, French Guiana; philippe.abboud@ch-cayenne.fr (P.A.); felix.djossou@ch-cayenne.fr (F.D.); loic.epelboin@ch-cayenne.fr (L.E.); 6 Laboratory, Centre Hospitalier Andree Rosemon Cayenne, 97300 Cayenne, French Guiana; magalie.demar@ch-cayenne.fr; 7 Unité Mixte de Recherche Tropical Biome and Immunopathology, Université de Guyane, 97300 Cayenne, French Guiana; 8 Service d’Anatomopathologie, Centre Hospitalier Andree Rosemon Cayenne, 97300 Cayenne, French Guiana; Kdrak.alsibai@doctor.com; 9 Service de Médecine, Centre Hospitalier de l’Ouest Guyanais, 97320 Saint Laurent du Maroni, French Guiana; c.misslin-tritsch@ch-ouestguyane.fr; 10 Département d’Information Médicale, Centre Hospitalier de l’Ouest Guyanais, 97320 Saint Laurent du Maroni, French Guiana; b.ntab@ch-ouestguyane.fr

**Keywords:** disseminated histoplasmosis, HIV, diagnosis, clinical presentation, polymorphism, French Guiana

## Abstract

We aimed to describe the ways patients with disseminated histoplasmosis—a multifaceted and often lethal disease—present themselves and are explored. A retrospective, observational, multicentric study spanned the period between 1 January 1981 and 1 October 2014. Principal component analysis was performed for the sampling sites and for the clinical signs and symptoms. The factor loadings of the principal components were selected for eigenvalues > 1. The most frequent signs and symptoms were an alteration of the WHO general performance status, fever, digestive tract, respiratory signs and symptoms and lymphadenopathies. The most common sites sampled were bone marrow, respiratory tract, blood, lymph node and liver biopsies, with significant variations in the number of sites from which samples were taken to try to identify the pathogen. The principal component analysis clinical signs and symptoms leading to the diagnosis showed four main lines of variation. The factor loadings of the four main components were compatible with four broad types of clinical presentations and four types of exploration strategies. Extracting simple algorithms was difficult, emphasizing the importance of clinical expertise when diagnosis depends on obtaining a sample where Histoplasma can be seen or grown. *Histoplasma* antigen detection tests will help simplifying the algorithms.

## 1. Introduction

Since the first description of the acquired immunodeficiency syndrome (AIDS) and the discovery of the virus, evidence-based medicine has led to remarkable progress in the care and treatment of HIV-infected patients [1]. Standardization of clinical trial endpoints has allowed cumulative progress despite the large number of trials. The knowledge gained from this research has been distilled and regularly updated through clinical guidelines for the care of patients [2,3,4]. The ramification of treatment groups in research protocols is echoed in clinical algorithms for clinicians. In French Guiana, a French overseas territory between Brazil and Suriname, the HIV epidemic has been evolving since the early 1980s. As a French territory, French Guiana has had access to diagnostic tests and treatments available in mainland France, and has been following the regular updates in the French National expert recommendations for HIV [5]. However, the Caribbean and South American connections, and the Amazonian pathogen ecosystem has led to specificities that are not always included in the National Guidelines [6,7,8]. Perhaps one of the greatest particularities is the high incidence of disseminated histoplasmosis, the main AIDS-defining infection in French Guiana [9,10]. This awareness has been present since the 1980s, starting with the dermatologists, then growing to all physicians as fungal culture was implemented and efforts and experience in identifying the fungal pathogen grew [11,12]. Disseminated histoplasmosis was hardly mentioned in the French recommendations for a long time, and it was absent from many National guidelines, or international strategic plans [13,14]. This lack of awareness about histoplasmosis and the lack of availability of diagnostic methods led to thousands of annual deaths in Latin America [15]. In consequence, using the GRADE approach (Grading of Recommendations, Assessment, Development, and Evaluation), the Pan American Health Organization (PAHO) has endeavored to develop guidelines for the diagnosis and treatment of disseminated histoplasmosis, guidelines that were published in 2020 with the hope that patients with advanced HIV will benefit from evidence-based algorithms [16]. In this context, we aimed to describe our clinical experience in French Guiana, notably to determine if there were some standard practices in the ways patients with disseminated histoplasmosis—a multifaceted disease—are explored. From a broad and detailed data collection, we used impartial methods to reduce the dimensionality of data into clinical and paraclinical categories of relevance for clinicians.

## 2. Methods

### 2.1. Study Design

The study was retrospective, observational, multicentric and concerned the period between 1 January 1981 and 1 October 2014.

### 2.2. Study Population

The study population was co-infections with HIV and histoplasmosis included in the Histoplasmosis and HIV database of French Guiana. Inclusion criteria were age >18 years, confirmed HIV infection, first proven episode of histoplasmosis (either by direct mycological examination, culture mycological or histological examination (excluding PCR) performed on a variety of different samples (plasma, myelogram, digestive biopsies, skin biopsies, bronchoalveolar lavage, etc.)) following EORTC/MSG criteria (European Platform of Cancer Research/Mycoses Study Group) [17]. HIV-infected patients with suspicion of infection also benefitted from thorough investigations searching for other fungi, parasites, bacteria, or viruses.

Unproven histoplasmosis (successful empirical antifungal therapy) or diagnosis based solely on the positivity of PCR, or recurrent histoplasmosis was not considered.

### 2.3. Study Conduct

This HIV–Histoplasmosis database was created in 1992. Incident cases of histoplasmosis in HIV-infected patients in the three hospitals of French Guiana (Cayenne, Kourou, and Saint Laurent du Maroni) were included. Epidemiological, clinical, paraclinical, immunovirological and therapeutic data were collected until October 2014 on a standardized paper form then entered into the database. Incident episodes of histoplasmosis in HIV-infected patients, previously known to be HIV positive or concomitantly discovered, and hospitalized in one of the three above hospitals, were included. The recorded variables were: socio-demographic data: sex, age, place of birth; clinical data: symptoms on admission, clinical entrance examination; immunovirological assessment, standard biological examinations; medical imaging, mycology, pathology.

### 2.4. Statistical Analysis

The statistical analysis was performed with STATA© and heatmaps were created with MS Excel. The number of investigated organ sites was computed for each patient and correlation matrices were generated and represented using heatmaps to improve their readability. Principal component analysis (PCA) is a common mathematical technique used for reducing the dimensionality of data whilst keeping as much variation as possible. It is frequently used in many areas but to our knowledge, never before in disseminated histoplasmosis, a disease that presents itself in different ways, and where microbiologic diagnosis requires fluid or tissue samples from affected organs. Principal component analysis was performed for the sampling sites and for the clinical signs and symptoms to try to estimate the dimensions of variance; the underlying reasoning would be that there are different presentations of histoplasmosis that lead to different paraclinical exploration priorities. Scree plots were performed, and the principal components were selected according to the Kaiser rule, which implies to drop all components with eigenvalues <1, 1 corresponding to the information accounted for by an average single item. The main principal components obtained following the Kaiser rule were subjected to the varimax orthogonal rotation and correlations of absolute values <0.3 were not shown to increase readability of factor loadings. The varimax rotation maximizes the sum of the variances of the squared factor loadings; it is a commonly used method to simplify the expression of a particular sub-space in terms of just a few major items each. For the present study, we used it to distill major presentation types from the breadth of clinical and paraclinical data. The Kaiser–Meyer–Olkin measure of sampling adequacy was used and was 0.51 for both sampling sites and clinical presentation.

### 2.5. Ethical and Regulatory Aspects

The 1992 Histoplasmosis and HIV anonymized database was approved by the Commission Nationale Informatique et Libertés (CNIL) (n° JZU0048856X, 07/16/2010), the French National Institute of Health and Medical Research institutional review board (CEEI INSERM) (IRB0000388, FWA00005831 18/05/2010), and by the Comité Consultatif pour le Traitement de l’Information pour la Recherche en Santé (CCTIRS) (N° 10.175bis, 10/06/2010).

## 3. Results

Overall, there were 349 observations of HIV-associated disseminated histoplasmosis. Before 1998 (year of fungal culture implementation), there were 47 cases of disseminated histoplasmosis; between 1998 and 2003, there were 100 cases of disseminated histoplasmosis; between 2004 and 2009, there were 11 cases of disseminated histoplasmosis; between 2010 and 2014, there were 91 cases of disseminated histoplasmosis. The mean age was 40 years (SD 9.7 years). The sex ratio was 1.9 (male/female). Patients had been in French Guiana for an average 23.9 years (SD = 16.5 years), 33 years for French (SD 14.8 years) and 11.5 years for foreign patients (SD 8.6 years). The median CD4 count was 31 per mm^3^ (IQR = 12–70). The median CD4 count was significant in patients with fever (30(IQR = 11–63) vs 66(IQR = 23–126) in those without fever, *p* = 0.001), in those with a WHO performance score >2, (25(IQR = 9–51) vs. 39(IQR = 17–88) in those with a score <3, *p* < 0.001) in those with pulmonary symptoms 25(IQR = 10–49) vs. 42(IQR = 16–85) in those without pulmonary symptoms, *p* < 0.001).

The most frequent signs and symptoms were an alteration of the WHO general performance status, fever, digestive tract, respiratory signs and symptoms and lymphadenopathies (Table 1). The most common sites sampled were bone marrow, respiratory tract, blood, lymph node and liver biopsies (Table 1). There were variations in the number of sites from which samples were taken to try to identify the pathogen.

When looking at the principal component analysis clinical signs and symptoms leading to the diagnosis there were four main lines of variation explaining 62% of the variation. The factor loadings of the four main components (eigenvectors) were compatible with four broad types of clinical presentations. When looking at the principal component analysis sampling sites investigated to identify the pathogen there were also four main dimensions. There again, there were four main components that explained 56% of the variation. After varimax rotations, the loadings greater than 0.3 for the first four principal components are shown in Table 2 for clinical presentation and in Table 3 for sampling sites to identify the infecting pathogen. For clinical aspects, the first eigenvector captured weight loss, cutaneomucous lesions, and digestive symptoms; the factor loadings of the second eigenvector captured fever and weight loss; the factor loadings of the third eigenvector captured lymph nodes, neurological symptoms and oral signs; finally, the factor loadings of the fourth eigenvector captured alteration of the general condition and pulmonary symptoms. Broadly this represented four stereotypic presentations: digestive presentation, fever-only syndrome, the enlarged lymph nodes and the pulmonary presentation.

Table 4 and Table 5 show the correlation matrices between different signs and symptoms leading to the current hospitalization, and the sites being sampled to try to identify the pathogen. There were statistically significant positive pairwise correlations between the following clinical signs and symptoms (Table 4): alteration of the general condition and weight loss, alteration of the general condition and fever, alteration of the general condition and neurological presentation, the duration of fever and alteration of the general condition and a pulmonary presentation, ocular and cutaneous presentation. There were statistically significant negative pairwise correlations between the following clinical signs and symptoms: alteration of the general condition and oral presentation, a digestive presentation and the duration of weight loss, a digestive presentation and oral lesions. There were statistically significant positive pairwise correlations between sample collection sites (Table 5): urine and cerebrospinal fluid, bone marrow and cerebrospinal fluid, upper digestive and urine, blood and urine. There were statistically significant negative pairwise correlations between sample collection sites (Table 5): upper digestive and lymph nodes, lower digestive and lymph nodes, upper digestive and cerebro-spinal fluid, lower digestive and bone marrow, lower digestive and bronchoalveolar, blood and liver.

## 4. Discussion

The present results show a great heterogeneity in the way patients with confirmed disseminated histoplasmosis were investigated. Physicians have a very good knowledge and experience of histoplasmosis, yet there was great variability in management, suggesting that physicians are very clinical in the way they approach an HIV-infected patient with a suspected opportunistic infection. Whereas outpatient care is highly protocolized with clear algorithms, in-hospital care is less so, presumably because of the combinatorial complexity of all the potential differential diagnoses that must be ruled out in an immunocompromised patient. Such complexity is much harder to break down in simple algorithms. For histoplasmosis, this is especially the case when the diagnosis of disseminated histoplasmosis relies on identifying Histoplasma at one of the infected sites.

Hence, this complexity is hard to simplify into crisp guidelines. The arrival of *Histoplasma* antigen detection tests will presumably homogenize the paraclinical algorithms to diagnose histoplasmosis, making them easier to implement.

Disseminated histoplasmosis is defined by the spread of histoplasma infection to more than one site. Initially the pathogen is usually inhaled and then spreads from the lungs via infected macrophages that disseminated to other organs. However, the reason why some patients will have predominant pulmonary, lymph node, hepatic, intestinal, cutaneous, medullar focalization remains obscure. Perhaps there is a random element, there are explanations linked to the circulation of immune cells within the body, to subtle differences in immune defenses within the host, or perhaps inflammation sites may recruit infected macrophages and lead to dissemination within other organs.

Overall, the four main sources of variability could be related to four major “typical” presentations distilled from the proteiform expression of disseminated histoplasmosis: first, a patient with a digestive presentation; second, patients with fever, general condition impairment but no focal signs; third, patients with enlarged lymph nodes; fourth, patients with a pulmonary presentation.

This is non-limitative but covers the most frequent situations. Each of these four typical presentations usually elicits the search for distinct differential diagnoses: the first would be, before endoscopy, intestinal pathogens; the second, typical mycobacterial infections; the third, tuberculosis and lymphoma; the fourth, tuberculosis, pneumocystosis and bacterial pneumonia.

Hence, this complexity requires great expertise and is hard to simplify into crisp guidelines. The arrival of *Histoplasma* antigen detection tests will presumably homogenize the paraclinical algorithms to diagnose histoplasmosis, making them easier to implement, notably in areas where experienced infectious diseases physicians are rare.

In conclusion, the analysis of 34 years of clinical experience of disseminated histoplasmosis underlines that it may present in different ways. However, it could be broken down into four main presentations which all have specific differential diagnoses. Despite these efforts to simplify the clinical variation, it remains difficult to extract simple algorithms and clinical expertise seems particularly important to reach the diagnosis when it depends on obtaining a sample where Histoplasma can be seen or grown. *Histoplasma* antigen detection tests will be easier to implement and systematic blood and or urine screening in an immunocompromised patients will surely use a much simpler algorithm.

## Figures and Tables

**Table 1:** Frequency of different signs and symptoms and sites sampled to try to identify the pathogen.

Variable	N	Proportion (%)	Standard Deviation
**Signs and Symptoms**			
Fever	348	89	31
Weigh loss	158	83	37
Digestive	348	70	45
Lymph node	348	48	50
Pulmonary	348	47	50
Neurological	348	20	40
Cutaneous	348	6	25
Oral	348	5	22
Ocular	348	0.28	5
**Sampling Sites**			
Bone marrow	349	61	48
Alveolobronchial	349	28	45
Blood	349	26	43
Liver	349	24	43
Lymph node	349	20	40
Lower digestive	349	19	39
Cerebro-spinal fluid	349	17	38
Cutaneomucous	349	11	31
Upper digestive	349	8	28
Urine	349	5	23

**Table 2:** Factor loadings with correlations >[0.3] for the four main principal components for clinical signs and symptoms leading to diagnosis.

Variable	Principal Component 1	Principal Component 2	Principal Component 3	Principal Component 4	Proportion of Variance Unexplained (%)
Altered general condition				0.52	0.43
Duration weight loss	0.37	0.40			0.42
Duration fever		0.90			0.17
Respiratory symptoms				−0.72	0.31
Cutaneous symptoms	0.44				0.64
Oral symptoms	0.50		−0.32		0.33
Lymphadenopathies			0.66		0.32
Digestive symptoms	−0.61				0.34
Neurological symptoms			0.55		0.45

Legend: The first component captured weight loss, cutaneomucous lesions, and digestive symptoms; the factor loadings of the second component captured fever and weight loss; the factor loadings of the third component captured lymph nodes, neurological symptoms and oral signs; finally, the factor loadings of the fourth principal component captured alteration of the general condition and pulmonary symptoms. Broadly this represented four stereotypic presentations: digestive presentation, fever-only syndrome, the enlarged lymph nodes and the pulmonary presentation.

**Table 3:** Factor loadings with correlations >[0.3] for the four main principal components for sampling sites to identify the infecting pathogen.

Variable	Principal Component 1	Principal Component 2	Principal Component 3	Principal Component 4	Proportion of Variance Unexplained (%)
Lymph nodes				−0.74	0.32
Cerebrospinal		0.65			0.41
Urinary	0.57				0.42
Upper digestive tract	0.44				0.57
Lower digestive tract		−0.33		0.40	0.36
Liver	−0.32			0.35	0.64
Blood	0.47				0.55
Cutaneomucous			0.65		0.38
Bone marrow		0.59			0.49
Bronchoalveolar			0.64		0.42

Legend: The first component captured liver, upper digestive, blood and urine sampling; the factor loadings of the second component captured bone marrow, lower digestive tract and cerebrospinal fluid; the factor loadings of the third component captured cutaneomucous and bronchoalveolar; finally, the factor loadings of the fourth principal component captured lymph nodes, lower digestive tract and liver.

**Table 4:** Pairwise correlation matrix different signs and symptoms leading to the current hospitalization.

	Alteration General Condition	Weight Loss	Weight Loss Duration	Fever	Days Fever	Pulmonary Symptoms	Cutaneous Symptoms	Oral Symptoms	Lymph Nodes	Digestive Symptoms	Neurological Symptoms	Occular Symptoms
alteration general condition	1											
weight loss	0.17	1										
weight loss duration	−0.03		1									
fever	0.21	0.05	−0.03	1								
days fever	0	0.15	0.31		1							
pulmonary symptoms	0.09	0.12	0.04	0.17	0.02	1						
cutaneous symptoms	−0.07	0.03	0.04	−0.05	0.1	0.1	1					
oral symptoms	−0.14	−0.04	0.09	−0.08	0.02	0.02	0.08	1				
lymph nodes	−0.04	0.11	−0.02	−0.05	−0.06	0.08	−0.06	−0.06	1			
digestive symptoms	0.01	−0.08	−0.22	0.06	0.03	−0.07	−0.1	−0.2	−0.05	1		
neurological symptoms	0.14	0.06	−0.02	−0.01	0.1	0.01	0.03	−0.03	−0.03	0.04	1	
ocular symptoms	−0.03			0.02		−0.05	0.2	−0.01	−0.05	−0.08	−0.03	1

Statistically significant correlations between clinical signs and symptoms: (a) Positive correlations are presented in green shades, with densities proportional to color hue. There was a positive correlation between alteration of the general condition and weight loss, alteration of the general condition and fever, alteration of the general condition and neurological presentation, duration of fever and duration of weight loss, fever and pulmonary presentation, ocular and cutaneous presentation; (b) Negative correlations are presented in pink/red shades, with densities proportional to color hue. There was a negative correlation between alteration of the general condition and oral presentation, digestive presentation and duration of weight loss, digestive presentation and oral lesions.

**Table 5:** Pairwise correlation matrix between sites being sampled to try to identify the pathogen.

	Lymph Node	Spinal Fluid	Urine	Upper Digestive	Lower Digestive	Liver	Blood	Bone Marrow	BAL
lymphnode	1								
spinal fluid	−0.04	1							
urine	0	0.18	1						
upper digestive	−0.11	−0.04	0.27	1					
lower digestive	−0.19	−0.11	0.19	0.21	1				
liver	−0.05	0.02	−0.06	−0.09	−0.04	1			
blood	0.03	0.07	0.22	0.11	0.03	−0.11	1		
bone marrow	−0.04	0.15	0.02	−0.02	−0.13	0.03	−0.02	1	
BAL	−0.02	0.03	−0.07	−0.06	−0.2	0.02	−0.06	−0.05	1

Statistically significant correlations between sample collection sites: (a) Positive correlations are presented in green shades, with densities proportional to color hue. There was a positive correlation between urine and csf, bone marrow and cerebro spinal fluid, upper digestive and urine, lower digestive and urine, blood and urine; (b) Negative correlations are presented in pink/red shades, with densities proportional to color hue. There was a negative correlation between upper digestive and lymph nodes, lower digestive and lymph nodes, upper digestive and csf, lower digestive and bone marrow, lower digestive and Bronchoaleveolar lavage, blood and liver.
